# Bioproduction of succinic acid from xylose by engineered *Yarrowia lipolytica* without pH control

**DOI:** 10.1186/s13068-020-01747-3

**Published:** 2020-06-27

**Authors:** Ashish A. Prabhu, Rodrigo Ledesma-Amaro, Carol Sze Ki Lin, Frederic Coulon, Vijay Kumar Thakur, Vinod Kumar

**Affiliations:** 1 School of Water, Energy and Environment, Cranfield University, Cranfield, MK43 0AL UK; 2 Department of Bioengineering and Imperial College Centre for Synthetic Biology, Imperial College London, London, SW7 2AZ UK; 3 School of Energy and Environment, City University of Hong Kong, Kowloon Tong, Kowloon, Hong Kong; 4 Biorefining and Advanced Materials Research Centre, Scotland’s Rural College (SRUC), Edinburgh, UK

**Keywords:** Xylose, *Yarrowia lipolytica*, Succinic acid, Acetic acid, pH

## Abstract

**Background:**

Xylose is the most prevalent sugar available in hemicellulose fraction of lignocellulosic biomass (LCB) and of great interest for the green economy. Unfortunately, most of the cell factories cannot inherently metabolize xylose as sole carbon source. *Yarrowia lipolytica* is a non-conventional yeast that produces industrially important metabolites. The yeast is able to metabolize a large variety of substrates including both hydrophilic and hydrophobic carbon sources. However, *Y. lipolytica* lacks effective metabolic pathway for xylose uptake and only scarce information is available on utilization of xylose. For the economica feasibility of LCB-based biorefineries, effective utilization of both pentose and hexose sugars is obligatory.

**Results:**

In the present study, succinic acid (SA) production from xylose by *Y.* *lipolytica* was examined. To this end, *Y. lipolytica* PSA02004 strain was engineered by overexpressing pentose pathway cassette comprising xylose reductase (*XR*), xylitol dehydrogenase (*XDH*) and xylulose kinase (*XK*) gene. The recombinant strain exhibited a robust growth on xylose as sole carbon source and produced substantial amount of SA. The inhibition of cell growth and SA formation was observed above 60 g/L xylose concentration. The batch cultivation of the recombinant strain in a bioreactor resulted in a maximum biomass concentration of 7.3 g/L and SA titer of 11.2 g/L with the yield of 0.19 g/g. Similar results in terms of cell growth and SA production were obtained with xylose-rich hydrolysate derived from sugarcane bagasse. The fed-batch fermentation yielded biomass concentration of 11.8 g/L (OD_600_: 56.1) and SA titer of 22.3 g/L with a gradual decrease in pH below 4.0. Acetic acid was obtained as a main by-product in all the fermentations.

**Conclusion:**

The recombinant strain displayed potential for bioconversion of xylose to SA. Further, this study provided a new insight on conversion of lignocellulosic biomass into value-added products. To the best of our knowledge, this is the first study on SA production by *Y.* *lipolytica* using xylose as a sole carbon source. 
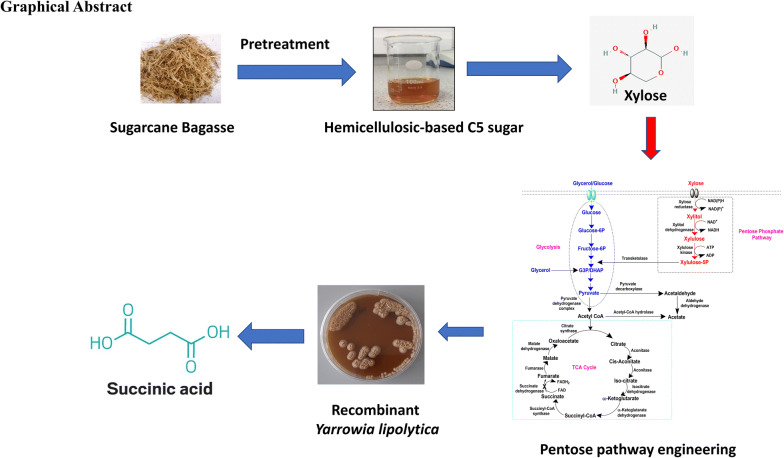

## Background

Microbial conversion of renewable biomass such as lignocellulosic feedstock into value-added products is getting a humongous response as it can replace the dependency on petroleum-based refineries, majorly responsible for rapid climate change and greenhouse gas emission [1]. Lignocellulosic biomass (LCB) is an abundant and rich source of renewable carbon and considered as a prominent feedstock to produce chemical commodities. LCB is a three-dimensional polymeric material composed of cellulose, hemicellulose and lignin. Cellulose is a linear homo-polymer of d-glucose while hemicellulose is a hetero-polymer. Xylose is the predominant sugar in hemicellulose and can constitute up to 30–40% of LCB [2]. Most of the studies are however focused on the utilization of cellulose for manufacturing value-added products, while the hemicellulosic portion is usually discarded as most microbes lack an efficient pathway for utilization of pentose sugar. In addition, carbon catabolite repression suppresses the assimilation of pentose sugars including xylose [3]. Therefore, efficient bioconversion of xylose is a prerequisite for economic feasibility of lignocellulosic biorefineries. Hence, more attention is paid to the rewiring of metabolic networks of microbial strains to utilize multiple carbon sources simultaneously, especially glucose and xylose, from the feedstock which will be essential for de-risking the commercial viability of the bioprocesses [2, 4].

Succinic acid (SA) (C_4_H_6_O_4_), is one of the 12 high-value bio-based chemicals listed by the US Department of Energy, and it has wide range of industrial applications [5]. Due to its versatility, the global market of SA is expanding with a demand of 50,000 metric ton in 2016 which is expected to double by 2025 [6, 7]. The chemical routes for SA synthesis are not only unsustainable, but they also suffer from reduced yield and low purity of the main product. As a result, in recent years, there is a growing interest towards creating a cleaner and greener technology for SA production and paradigm shift from petrochemical synthesis towards bio-based production of SA [8]. Currently, the bio-based production of SA constitute a significant fraction of the total market [9]. Despite the high potential, the growth of bio-based SA production witnessed a declining trend in recent years. Due to low petroleum prices, fossil-based SA production is cheaper than SA synthesized through biological route. Therefore, it is imperative to cut down the cost to make it economically viable and the utilization of crude renewable resources from waste streams could substantially reduce the production cost of SA. It is envisaged that with the use of low-cost agricultural feedstock, bioproduction will soon replace the conventional petroleum-based process [9, 10].

The fermentative production of SA occurs through the reductive and/or oxidative TCA cycle, utilization of CO_2_ as co-substrate which leads to high CO_2_ sequestration potential [11]. The biological production of SA has been investigated using several bacterial strains such as *Mannheimia* *succiniciproducens, Actinobacillus succinogenes* and recombinant *Escherichia* *coli* as a potential host [12]. Bacteria are very sensitive towards low pH and require moderate pH for their growth resulting in large consumption of neutralizing agent [11]. Further at neutral pH, SA is obtained in the form of succinate salts which complicates the downstream processing, and all these add extra cost to bioprocess. On the other hand, yeasts are the potential host to produce organic acids as they are naturally adapted to grow under low pH [13]. *Yarrowia lipolytica* is an oleaginous, non-conventional, robust and industrially important yeast with GRAS status. Being an aerobic yeast, the flux of TCA cycle is very active in *Y. lipolytica* and plethora of reports are based on the production of TCA intermediates including SA by the yeast [14, 15]. Furthermore, *Y. lipolytica* has the amazing ability to grow perfectly well over a wide pH range without any significant change in growth parameters [16]. All these features render this yeast species as an attractive host to produce SA.

**Fig. 1:**
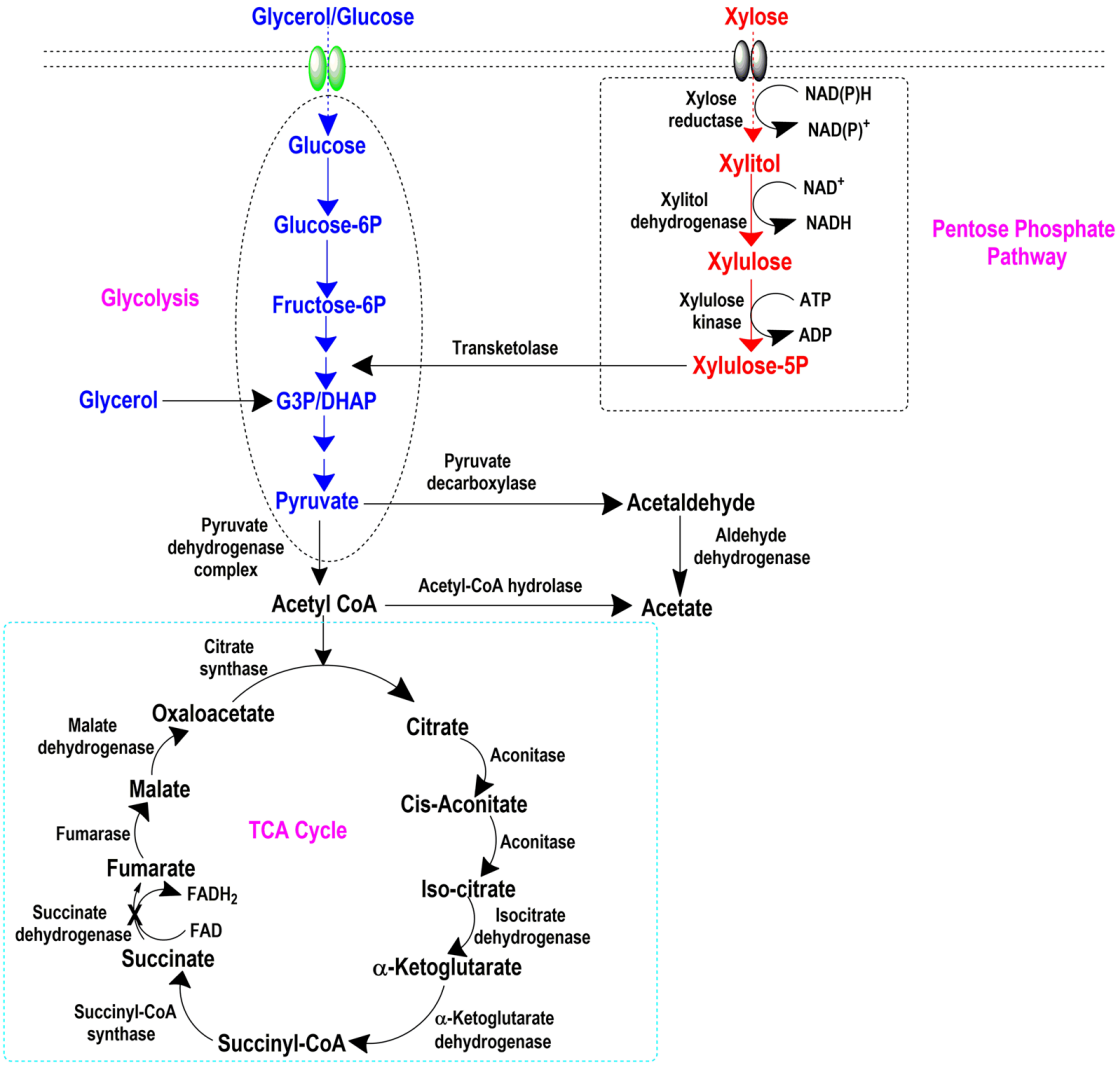
Metabolic pathway for SA production from different carbon sources by *Y. lipolytica* (*G3P* Glycerol-3-phosphate; *DHAP* Dihydroxyacetone phosphate). The introduced xylose pathway is highlighted in red color [6, 22]

Over the years, extensive efforts have been made to make it a superior host. Previously, *Y. lipolytica* has been engineered for production SA using glucose and glycerol as carbon sources [17, 18]. Most studies claimed that *Y. lipolytica* cannot naturally use xylose as the sole carbon source [19] and there is no report on xylose-based SA production by *Y. lipolytica*. In the present study, attempts were made to overexpress pentose pathway genes in *Y.* *lipolytica*. To this end, *Y. lipolytica* PSA02004 strain was engineered by overexpression of xylose reductase (*XR*), xylitol dehydrogenase (*XDH*) and xylulose kinase (*XK*) genes for efficient utilization of xylose and simultaneous production of SA (Fig. 1). The recombinant strain was grown on xylose, and the impact of co-substrates on cell growth and xylose metabolism were examined. To further visualize the maximum carbon uptake capability and product formation, the strain was subjected to different concentrations of xylose. The study was scaled up from shake flask to bioreactor with batch and fed-batch cultivations to further improve SA production. The engineered strain was also evaluated for SA production from xylose-rich hydrolysate derived from sugarcane bagasse (SCB). The pH was not controlled in any of the experiments carried out in this work in order to understand the robustness of the strain to withstand low pH conditions, without compromising the production. To our knowledge, this is the first report of SA production in *Y. lipolytica* with xylose as the sole carbon source.

## Results

### Shake flask cultivation of *Y. lipolytica* PSA02004

**Fig. 2:**
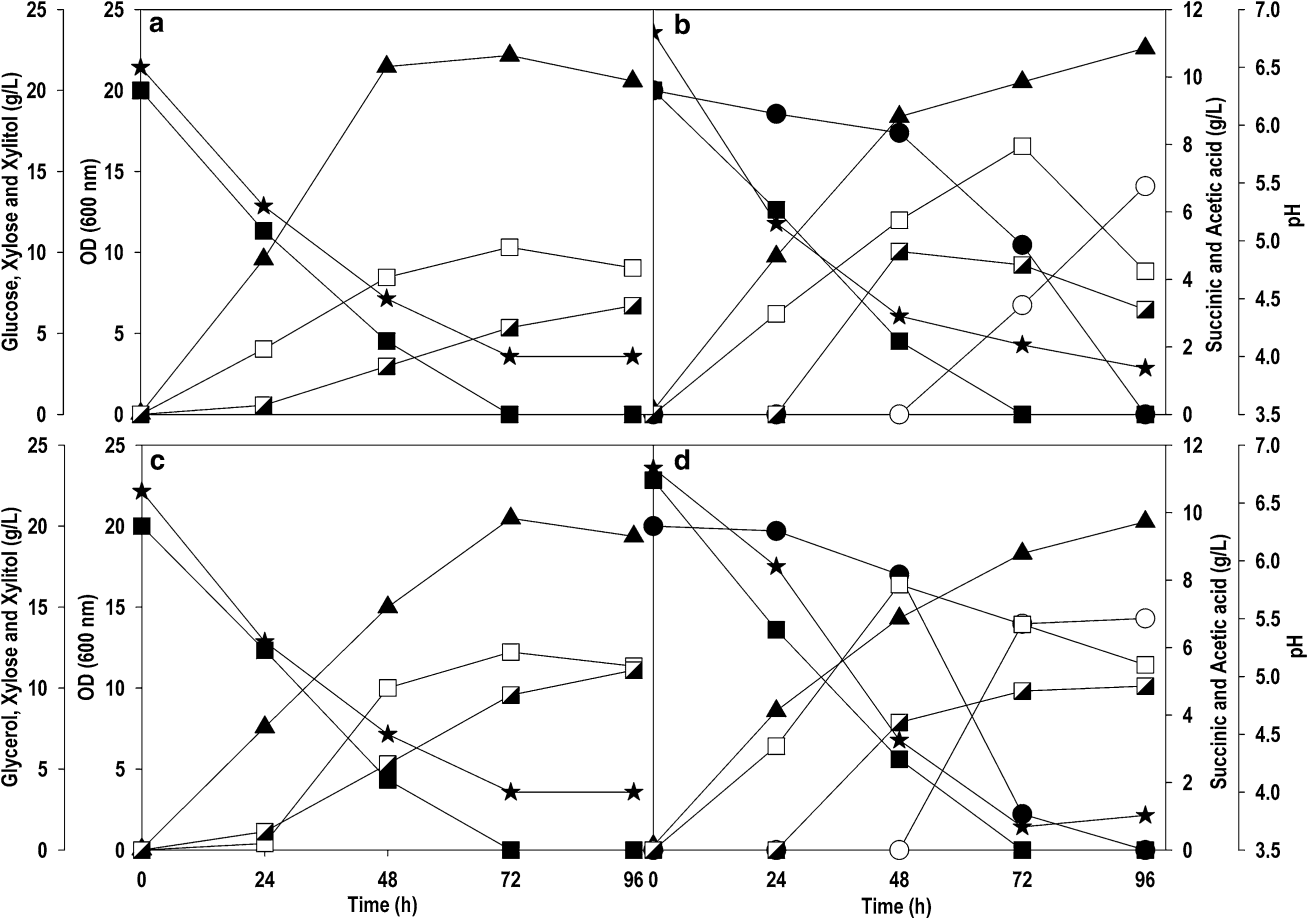
Time-course profiles of substrate consumption, OD_600_, pH, production of SA, AA and xylitol during shake flask culture of *Y. lipolytica* PSA02004 on **a** glucose, **b** glucose + xylose, **c** glycerol and **d** glycerol + xylose. Symbols: filled square (glucose or glycerol), filled triangle up (OD_600_), empty square (SA), semi-filled right square (AA), filled circle (xylose), empty circle (xylitol) and filled star (pH)

SA is an intermediate of TCA cycle and produced through oxidative/reductive TCA cycle [20]. The reductive pathway is not favorable thermodynamically and responsible for glucose repression. *Y. lipolytica* prefers to use oxidative TCA cycle for SA production [21, 22]. Succinate dehydrogenase is one of the enzymes of oxidative TCA cycle, which catalyzes the oxidation of succinate to fumarate and it has five subunits. Gao et al. inactivated *sdh5* encoding succinate dehydrogenase assembly factor 2 (*YALI0F11957g*) in Po1f strain (derived from W29 strain) and obtained a mutant PGC01003 [23], and this strain showed impaired growth on glucose. The PGC01003 strain was subjected to adaptive evolution using glucose-based medium for 21 days and the evolved strain was designated as *Y. lipolytica* PSA02004 [18]. This strain was cultured on glucose, glycerol, xylose, glucose/xylose and glycerol/xylose (Fig. 2). Glucose and glycerol are the preferred carbon sources for *Y. lipolytica*, and these carbon sources were completely depleted within 72 h concomitant with the cell growth, which also coincided with SA production (5.0-6.0 g/L) (Fig. 2a, c). However, the strain was unable to grow on xylose as the sole carbon source (data not shown). The co-fermentation of xylose with glucose or glycerol resulted in xylitol accumulation along with SA synthesis (6.5–8.0 g/L) (Fig. 2b, d) indicating that *Y.* *lipolytica* cannot metabolize xylose to grow on it, but it can transform xylose into xylitol with a high conversion yield (~ 70%). This was also supported by similar cell growth (OD_600_: 20–22) observed on glucose/glycerol, as well as during co-fermentation with xylose, where xylose was mainly utilized for xylitol synthesis and not contributing for biomass/product manufacturing. The xylose was subjected to carbon catabolite repression in the presence of glucose/glycerol, and rapid consumption of xylose along with xylitol accumulation started after 48 h when large fraction of these co-substrates was utilized. Acetic acid (AA) was obtained as a main by-product, which was evident in the late log phase of the cell growth, it can be correlated with subsequent drop in pH below 4.5 in all the fermentations. Another important observation was that the amount of SA and AA achieved during co-fermentation was marginally higher in comparison to fermentation on a single carbon source hinting at cryptic xylose metabolism in *Y. lipolytica.*

### Introduction of xylose metabolic pathway in *Y. lipolytica* PSA02004

**Fig. 3:**
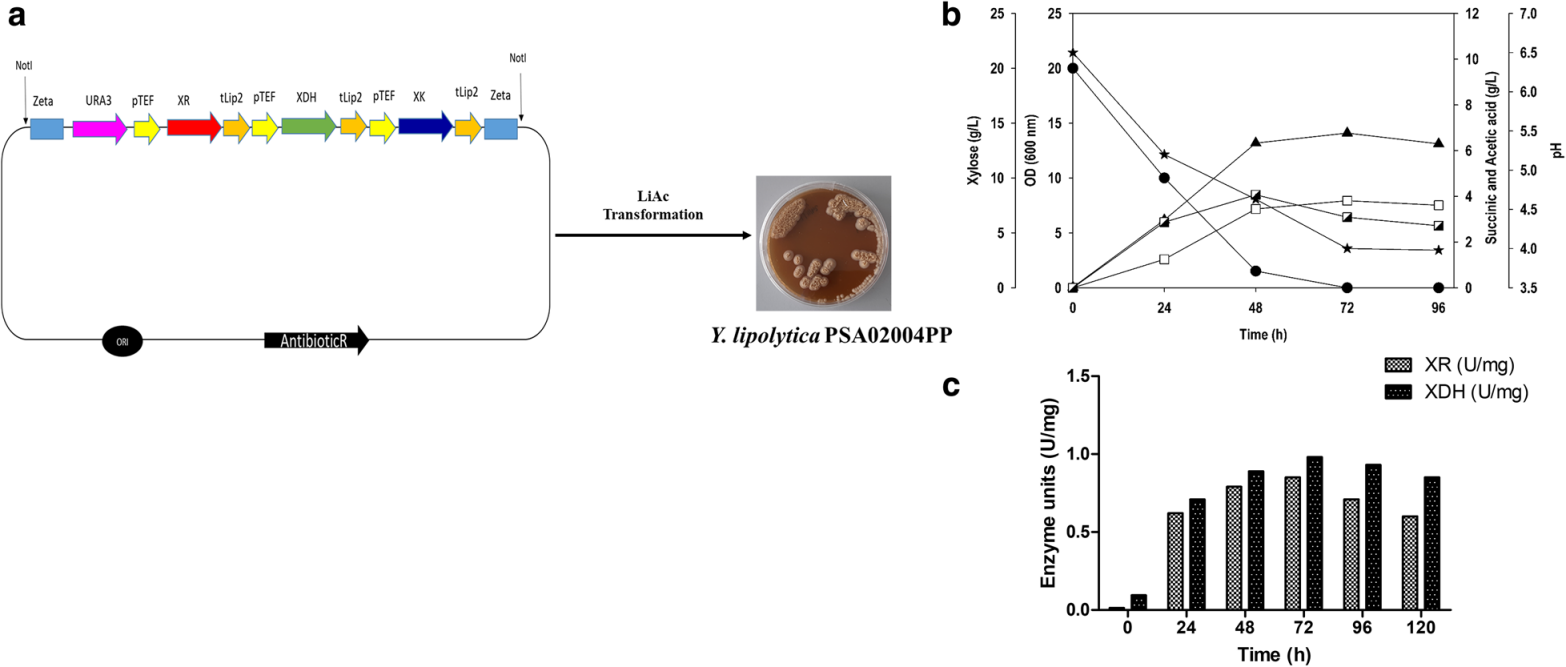
**a** Golden Gate Assembly bearing pentose pathway genes dedicated to integration with *Yarrowia lipolytica* PSA02004 genome; **b** time-course profiles of substrate consumption, OD_600_, pH, production of SA and AA during shake flask culture of *Y. lipolytica* PSA02004PP on xylose. Symbols: filled circle (xylose), filled triangle up (OD_600_), empty square (SA), semi-filled right square (AA) and filled star (pH); **c** XR and XDH activity profiles during shake flask cultivation of *Y. lipolytica* PSA02004PP

As shown in previous section, *Y. lipolytica* PSA02004 strain showed no growth on minimal medium supplemented with 20 g/L xylose as a sole carbon source. The inability of *Y. lipolytica* to assimilate xylose for cellular growth impedes its application for lignocellulosic biorefineries. To enable growth on xylose, xylose metabolic pathway was introduced in *Y. lipolytica* PSA02004. In this study, the engineered strain was constructed by overexpressing the homologous gene of xylose reductase (*XR*), xylitol dehydrogenase (*XDH*) and of xylulose kinase (*XK*) from *Y. lipolytica* (Po1d strain) cloned under transcription elongation factor (TEF) promoter (Fig. 3a). The resulting strain was designated as *Y.* *lipolytica* PSA02004PP. With the overexpression of *XR, XDH* and *XK*, the strain was able to grow in the medium containing xylose as a sole carbon source. The time course profiles of substrate assimilation, cell growth, product formation and pH were similar to those obtained on glucose or glycerol. There was no xylitol accumulation and probably, all the formed xylitol was funneled towards central carbon metabolism. The maximum OD_600_ obtained was 14.1 at 72 h. The recombinant strain PSA02004PP was able to produce 3.8 g/L SA from xylose with 0.19 g/g yield. Interestingly, substantial amount of AA (4.1 g/L) was accumulated. The combined production of two organic acids resulted in drop in pH with time (Fig. 3b). In addition to cultivation on xylose, the activity of two key enzymes, i.e., XR and XDH, involved in xylose metabolism was monitored throughout fermentation (Fig. 3c). The activity profiles revealed that high activities of XR and XDH were maintained during exponential growth and stationary phase. The maximum XR and XDH activity of 0.85 and 0.98 U/mg, respectively, were obtained at 72 h. The slightly high XDH activity than XR allows better synchronization between two enzymes, and results in efficient conversion xylose to xylulose without accumulation of xylitol as by-product.

### Co-fermentation of xylose with glucose/glycerol by *Y. lipolytica* PSA02004PP in shake flasks

**Fig. 4:**
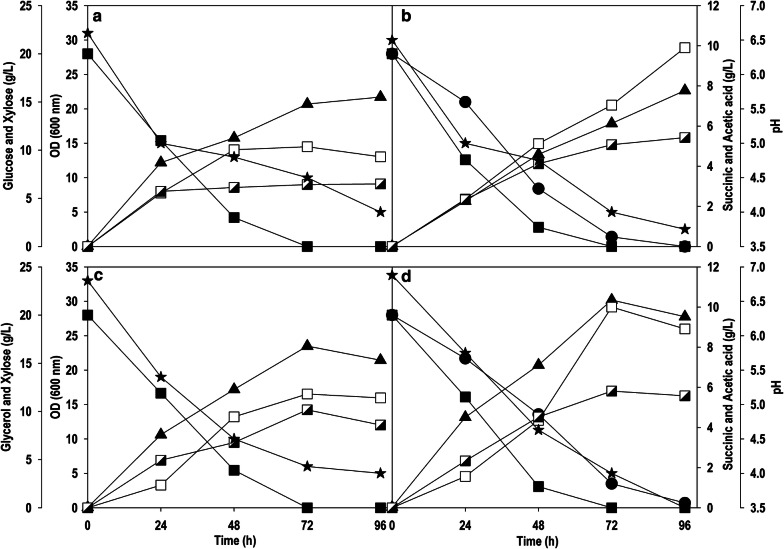
Time-course profiles of substrate consumption, OD_600_, pH, production of SA and AA during shake flask culture of *Y. lipolytica* PSA02004PP on: **a** glucose, **b** glucose + xylose, **c** glycerol and **d** glycerol + xylose. Symbols: filled square (glucose or glycerol), filled circle (xylose), filled triangle up (OD_600_), empty square (SA), semi-filled right square (AA) and filled star (pH)

The recombinant *Y. lipolytica* strain carrying a copy of *XR, XDH* and *XK* gene showed a superior growth characteristic along with SA synthesis in xylose containing medium under shake flask cultivation. Furthermore, the phenotypic profile on different carbon sources such as glycerol and glucose, and the effect of these substrates on the uptake of xylose were investigated. The recombinant strain produced SA titer of 5.7 and 5.0 g/L with glycerol and glucose as carbon source, respectively (Fig. 4a, c). While in case of co-fermentation with glucose or glycerol, the consumption of xylose was slowed down, indicating some signs of catabolite repression effect. The co-fermentation of glucose and xylose resulted in the maximum OD_600_ value of 22.7 with SA titer of 9.9 g/L at 96 h (Fig. 4b). While OD_600_ of 30.1 was achieved with similar resultant SA concentration (10.0 g/L) at a faster rate in 72 h using a mixture of glycerol and xylose (Fig. 4d). Additional accumulation of AA was observed both in individual sugars as well as with mixed substrates, which also reduced the pH of the fermentation broth besides reducing the SA yield. After the introduction of xylose metabolic pathway, no xylitol accumulation was observed with co-fermentations, and significant improvement in SA synthesis was noticed in comparison to control where xylose was transformed into xylitol in presence of glucose/glycerol. Thus, there was clear shift in metabolism with entry of xylose into central carbon metabolism.

### Substrate inhibition studies on recombinant *Y. lipolytica* PSA02004PP

**Fig. 5:**
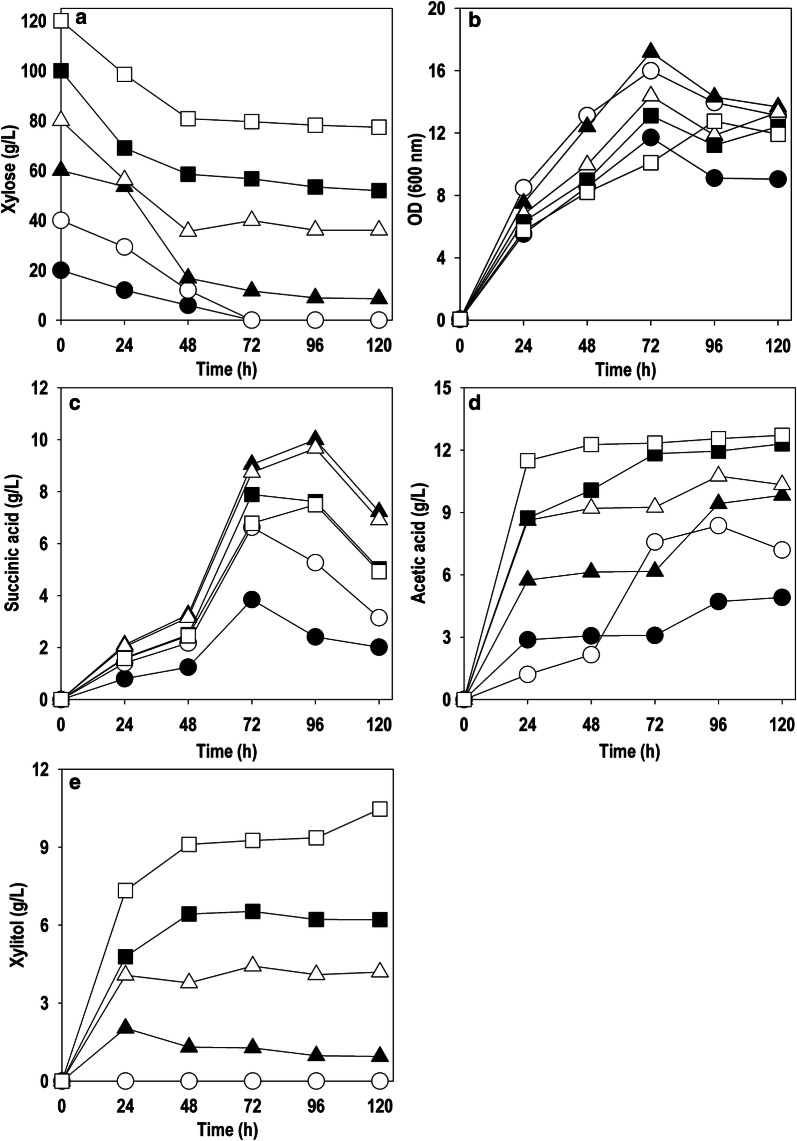
Time course profile of *Y. lipolytica* PSA02004PP at different xylose levels **a** substrate consumption, **b** OD_600_, **c** SA, **d** AA, **e** Xylitol. Symbols: filled circle (20 g/L), empty circle (40 g/L), filled triangle up (60 g/L), empty triangle up (80 g/L), filled square (100 g/L) and empty square (120 g/L)

The effects of initial xylose concentration on the substrate uptake rate, cell growth, product and by-product formation ability of *Y. lipolytica* PSA02004PP were investigated by growing the strain at different initial concentrations of xylose ranging from 20 to 120 g/L. The aim of the experiment was to determine optimal level of xylose for cell growth and SA production. Figure 5a–e shows the time course profiles for xylose uptake, cell growth (OD_600_), SA, AA and xylitol production. Xylose was completely consumed in 72 h for fermentation media with an initial level of 20 and 40 g/L. Beyond 40 g/L, residual xylose was noticed even at 120 h. The uptake of xylose was reduced after 48 h at 60, 80, 100 and 120 g/L. The amount of unutilized xylose at 60, 80, 100 and 120 g/L was 8.6, 36.1, 51.9 and 77.3 g/L, respectively (Fig. 5a). There was a linear increase in cell growth (i.e., OD_600_) from 11.7 to 17.2, as the initial xylose concentration was enhanced from 20 g/L to 60 g/L (Fig. 5b). Above 60 g/L, there was gradual decline in the biomass formation indicating the substrate inhibition. Similar trend was obtained with SA; producing a maximum of 3.8, 6.6 and 10.0 g/L at 20, 40 and 60 g/L initial xylose concentration, respectively (Fig. 5c). Further increase in initial xylose concentration retarded the yield and productivity of SA. AA was identified as the main by-product and accumulation enhanced at higher substrate concentration. The AA level reached 10–13 g/L at initial xylose concentration 80–120 g/L (Fig. 5d). Interestingly, xylitol formation was observed at xylose level above 40 g/L and significantly increased from 1.3 g/L to 10.5 g/L as initial xylose concentration was raised from 60 g/L to 120 g/L (Fig. 5e). The continuous increment in AA and xylitol production with increase in xylose levels can be due to overflow metabolism at higher substrate concentrations. The initial xylose concentration of 60 g/L was selected for further experiments to achieve an optimal balance between SA titer, yield and productivity.

### Batch cultivation of *Y. lipolytica* PSA02004PP in bench-top-scale bioreactor

**Fig. 6:**
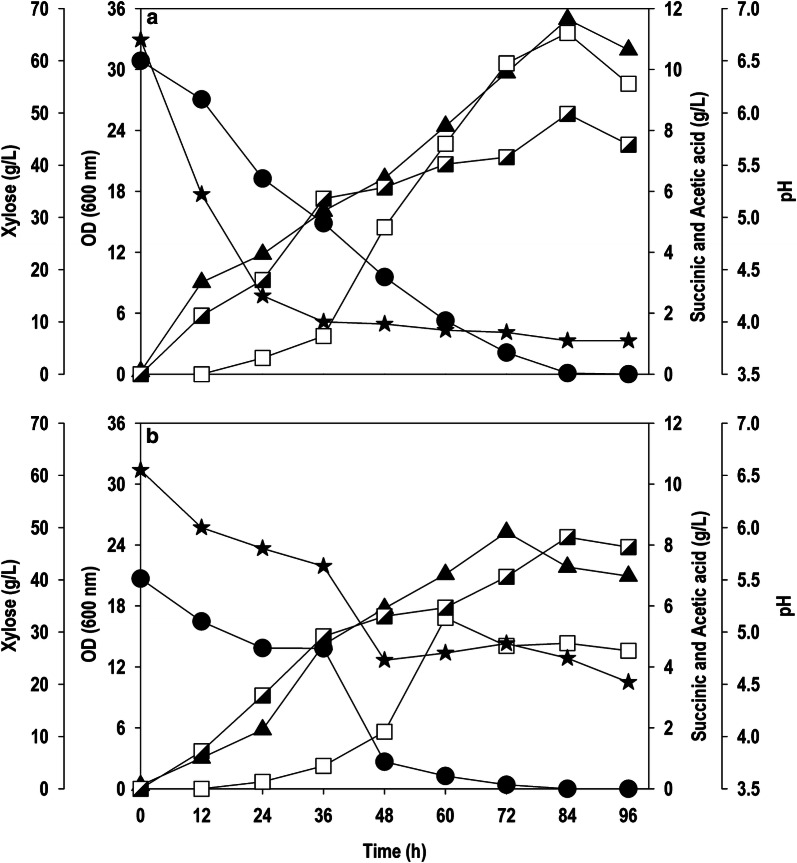
Batch cultivation of *Y. lipolytica* PSA02004PP in bioreactor using **a** pure xylose, **b** xylose-rich hydrolysate derived from sugarcane bagasse. Symbols: filled circle (xylose), filled triangle up (OD_600_), empty square (SA), semi-filled right square (AA), and filled star (pH)

The batch cultivation of recombinant *Y. lipolytica* PSA02004PP was conducted in bench-top bioreactor in order to understand the phenotypic characteristic of strain. The initial concentration of pure xylose was 60 g/L. The strain was able to produce maximum biomass concentration of 7.3 g/L (OD_600_: 34.9) with pure xylose substrate (Fig. 6a). The xylose was almost completely consumed (> 99%) in 84 h, which was reflected in concomitant termination of biomass, SA and AA formation. The highest SA level of 11.2 g/L was obtained with the yield of 0.19 g/g and 8.5 g/L of AA was generated in the same duration. The experiment was repeated with crude xylose-rich hydrolysate derived from SCB (Fig. 6b). Hydrolysate after pre-treatment often contains inhibitors which can negatively impact the performance of microorganisms. The comparison was made to evaluate the robustness of strain in presence of fermentation inhibitor such as furfural and AA. The cell growth (OD_600_: 25.3; 5.3 g/L) was unaffected as biomass yield was almost the same in both cases. The strain accumulated 5.6 g/L SA with a yield of 0.14 g/g. In both fermentation, accumulation of substantial amount of AA (~ 8.5 g/L) along with SA resulted in significant reduction in pH. Furthermore, no accumulation of xylitol was observed during fermentation, indicating active pentose phosphate pathway resulted in enhanced biomass formation.

### Fed-batch fermentation for SA production

**Fig. 7:**
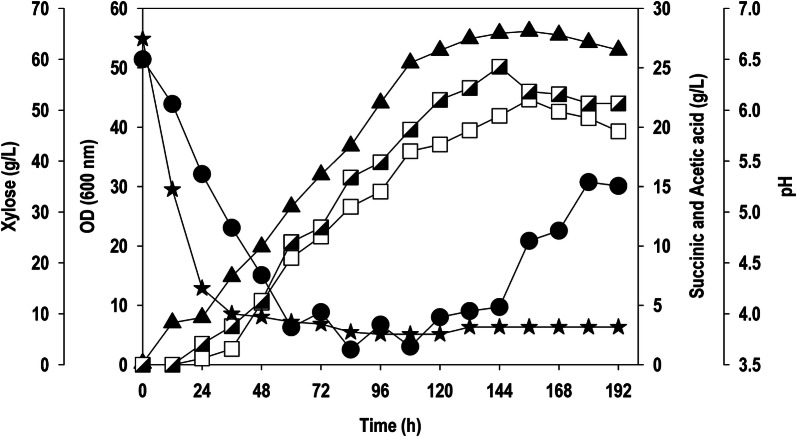
Fed-batch kinetics of xylose uptake, cell growth, product formation and pH during *Y.* *lipolytica* PSA02004PP fermentation in bioreactor. Symbols: filled circle (xylose), filled triangle up (OD_600_), empty square (SA), semi-filled right square (AA), and filled star (pH)

Based on the batch fermentation study, where the strain displayed excellent xylose uptake capability with simultaneous biosynthesis of SA, fed-batch fermentation was conducted to further improve SA production. The strain was evaluated in fed-batch fermentation with minimal medium without controlling pH. The batch phase was completed in 72 h of fermentation, where the initial xylose concentration was reduced to 10.3 g/L, and the strain was in exponential phase with a maximum OD_600_ of 32.0, which is equivalent to 6.7 g/L biomass concentration. The SA and AA concentration at 72 h were 10.8 g/L and 11.6 g/L, respectively. The cell metabolism coupled with accumulation of these organic acids caused reduction in pH level to 3.9 and thereafter, pH was stable till the end of fermentation. The feeding was started after 72 h to maintain a xylose level above 10 g/L (Fig. 7). The cell growth was continued till 108 h, thereafter, the cell reached stationary phase and remained stable (OD_600_: 50–56). Despite a low pH, synthesis of biomass and SA was continued with a smooth rate. The highest biomass concentration of 11.8 g/L was observed at 156 h of fermentation. The maximum SA concentration was 22.3 g/L, which coincided with the cell growth. The fermentation resulted in the buildup of 25.0 g/L AA, a major by-product which was obtained in higher amount than the desired product SA.

## Discussion

SA is a top platform chemical with multitude of industrial applications, which offers access to a wide range of products with huge commercial market. In the last two decades, significant research efforts have been devoted towards bio-based SA production. Majority of reports based on SA bioproduction are from prokaryotes with only few studies using xylose as carbon source. *A.* *succinogenes* is considered as one of the favorite candidates for SA production, due to its high acid secreting capabilities and it can uptake a wide range of sugars [9, 11]. Even with the higher productivity, the strain is associated with the constraints such as inability to grow at low pH and requirement of carboxylating agent for active reductive SA pathway, which makes it unsuitable for industrial level production.

*Y. lipolytica* is well-known for the accumulation of intracellular lipids and extracellular secretion of organic acids and polyols [15]. Also, the yeast has been explored for SA production from glycerol and glucose by our research groups [17, 24, 25]. Very recently, our groups reported SA production from co-fermentation of glucose and xylose by *Y. lipolytica* [22], in which pure as well as crude glucose and xylose from SCB were utilized as carbon sources. Glucose was completely consumed by the yeast; however, large fraction of xylose (50–70%) remained unutilized. The xylose utilization was repressed in the presence of glucose, which is in agreement with our results. Little is known about xylose metabolizing ability of *Y. lipolytica*, and there are few literature reports available on the uptake of xylose by *Y. lipolytica* which is quite contradictory. Majority of the studies indicates that *Y.* *lipolytica* shows a restricted uptake of xylose prior to adaptation or starvation periods. Genome mining of *Y. lipolytica* showed the presence of xylose pathway, however the quantitative polymerase chain reaction (qPCR) showed weak mRNA expression of XDH gene (*YALI0E12463*), indicating the hypothesis that weak expression of XDH is the limiting factor [26]. According to Rodriguez et al. [27], xylose pathway is present in the yeast, but it was poorly expressed due to cryptic genetic circuits controlling expression of the key enzymes. It was found that overexpression of endogenous XDH (*YALI0E12463*) and XK (*YALI0F10293*) in *Y. lipolytica* Po1f strain under the control of UAS1B8-TEF_min_ promoter resulted in cell growth on xylose. In the same year, Ledesma-Amaro et al. engineered *Y. lipolytica* by introducing XR and XDH from *S. stipitis*, which serves as a model organism for xylose metabolism [28]. They observed that overexpression of XR and XDH is insufficient to enable growth on xylose, and additional overexpression of XK allowed identical growth to wild-type strain. The engineered strain was able to produce lipids and citric acid utilizing xylose as a sole carbon source. The same group also demonstrated the utilization of xylose-rich agave bagasse hydrolysate by *Y. lipolytica* harboring *XR, XDH* and *XK* gene cassette under the control of TEF promoter [29].

In the present study, three enzymes, XR, XDH and XK, were overexpressed under the control of constitutive TEF promoter in *Y. lipolytica* PSA02004. The metabolic pathway for xylose utilization and SA production by recombinant *Y. lipolytica* PSA02004PP is depicted in Fig. 1. The control strain was unable to grow on xylose as the sole carbon source, but it was able to transform xylose to xylitol in the presence of co-substrates such as glucose and glycerol (Fig. 2). Similar results were obtained by Ledesma-Amaro et al. [28] and Prabhu et al. [30] where biotransformation of xylose into xylitol was observed during co-fermentations with glucose/glycerol. The introduction of xylose pathway resulted in a marked change in metabolism. The recombinant strain was able to grow (OD_600_: 14.1) on xylose as the sole carbon source, as well as accumulated SA (3.8 g/L), and generated AA (4.1 g/L) as a by-product (Fig. 3b). In case of co-fermentation with xylose and glucose/glycerol, the xylitol formation was not observed and SA production increased in comparison to the control strain, which is supported by the high XDH activity during the exponential growth phase (Fig. 4b, d). Walfridsson et al. [31] reported that high XDH to XR ratio resulted in no xylitol accumulation and high ethanol formation rate in recombinant *Saccharomyces cerevisiae* strain integrated with multi-copy gene of *xyl2* encoding xylitol dehydrogenase. The carbon catabolite repression was observed with xylose and its rapid consumption began after 48 h when glucose/glycerol was largely assimilated. This is in agreement with results obtained by Ledesma-Amaro et al. [28] and Ong et al. [22]. In the current study, we investigated the impact of initial xylose levels on *Y.* *lipolytica* using the minimal medium, and it was observed a substrate inhibition beyond 60 g/L. The xylose was completely utilized until 40 g/L and with further increase in the initial xylose concentration, the amount of residual xylose continuously elevated (Fig. 5a). The substrate inhibition phenomenon also negatively affected SA production (Fig. 5c). The xylitol accumulation was detected at higher xylose concentrations indicating overflow metabolism (Fig. 5e). XR and XDH enzymes are dependent on NAD(P)H and NAD^+^, respectively. At high concentration of xylose, redox imbalance will be provoked and results in xylitol accumulation [32]. Similar trend was observed by Ledesma-Amaro et al. [28]. In their study, xylose was completely consumed at up to 30 g/L. However, *Y.* *lipolytica* was unable to consume all the xylose at initial concentration 60–90 g/L, and 15–70% of xylose was left unconsumed at these concentrations along with xylitol accumulation. Similar pattern of substrate inhibition was observed by Salvachúa et al. [33] with *A. succinogenes* beyond 60 g/L xylose concentration. The strain resulted in maximum of 48 g/L SA with 80 g/L xylose concentration.

The scale-up of data from shake flask to bioreactor level improved SA synthesis from 3.8 g/L to 11.2 g/L (Fig. 6a). The results obtained with crude xylose from SCB hydrolysate were highly encouraging. The yeast grew robustly (OD_600_: 25.3) on crude xylose despite the presence of AA and furfural at significant levels (Fig. 6b), in accordance with previous reports on agave hydrolysates [29]. The resultant SA concentration was 5.6 g/L with a yield of 0.14 g/g, which is similar to those obtained with pure xylose (0.19 g/g). The fed-batch cultivation of recombinant strain further improved the performance and yielded a biomass and SA concentration of 11.8 and 22.3 g/L, respectively, at 156 h (Fig. 7). Besides cell growth and SA synthesis, AA was continuously accumulated up to 25.0 g/L. These results indicate that more carbon flux is diverted towards by-product formation resulting in reduced SA production, however the strain showed resistance at lower pH condition (< 4.0). Cui et al. [17] reported excessive production of AA in glycerol fermentation using *Y. lipolytica* PGC1003 strain. Hyperaccumulation of AA might be due to the imbalance between the flux of glycolysis and TCA cycle, which interrupts the cell growth and also affects the cell metabolism [34]. Two different approaches can be employed to curb AA formation: disrupting pathways leading to AA formation, and the second one is diverting AA towards SA production. The AA produced, an undesirable product, can be combined with xylose utilization for SA production and this co-fermentation will be beneficial for efficient utilization of lignocellulosic hydrolysate containing substantial amount of AA [35–37]. In order to understand the robustness of the recombinant *Y. lipolytica* PSA02004PP strain to withstand adverse condition such as low pH, the pH was not controlled in fermentations carried out in this study. The strain was able to grow and biosynthesize SA continuously even after significant reduction in pH, which shows its robustness and flexibility. The recovery and purification of SA is an obstacle for commercial production [8]. The advantage at lower pH is that most of the product fraction will be in acidic form (rather than dissociated form), resulting in simple and cost-effective downstream processes [38]. Table 1 shows SA production by different microorganisms using xylose as carbon source. The SA titers obtained in current work are comparable to the data available in literature, making it competitive; however, the yield and volumetric productivity were lower. It was very evident that most of the bacteria such as *A. succinogenes*, *B. succiniciproducens* and *E. coli* with inherited xylose uptake metabolism have shown better SA production capabilities using xylose as a sole carbon source. Our work did not make use of any alkali or pH regulators to control/maintain the pH unlike other reports in Table 1. Although the current SA production would not be practical for commercial use, these results still suggest great potential for this engineered *Y. lipolytica* strain in the production of SA from xylose.

**Table 1:** Xylose-based succinic acid production by various microorganisms

Organism	Fermentation mode	Feedstock (xylose fraction)	Other sugars	Xylose consumed (g/L)	Succinic acid			Main by-products (g/L)	Reference
					Titer (g/L)	Yield^a^ (g/g)	Productivity (g/L/h)		
*A. succinogenes* 130Z	Batch	SSL (72.6%)	Galactose (12.2%), glucose (10.9%), mannose (4.2%), arabinose (0.1%)	30.2	27.4	0.70	0.45	FA (8.8) AA (12.6)	[45]
*B. succiniciproducens* JF4016	Batch	SSL (72.6%)	Galactose (12.2%), glucose (10.9%), mannose (4.2%), arabinose (0.1%)	25.0	26.0	0.76	0.55	FA (3.3) AA (8.3)	[45]
*A. succinogenes* 130Z	Batch	Pure xylose	–	22.00	14.2	0.64	0.67	–	[46]
*A. succinogenes* 130Z	Batch	SCB (-)	–	52.00	22.5	0.43	1.01	–	[46]
*E. coli* recombinant SD121	Batch	Pretreated mother liquor (51.7%)	Arabinose (10–15%), glucose (8–10%), galactose (8–10%)	37.01	52.1	0.63	0.62	AA (10)	[47]
*A. succinogenes* CICC 11014	Batch	Corncob hydrolysate (77.3%)	Glucose (2.8%), arabinose (12.9%), cellobiose (7.0%)	38.1	23.64	0.58	0.49	–	[48]
*E. coli* BA204	Batch dual phase	Pretreated cornstalk (80.7%)	Glucose (13.5%)	8.1	11.13	1.03	0.70	AA (2.5)	[49]
*E. coli* BA408	Batch	Corn stalk hydrolysate (81.6%)	Glucose (9.5%), arabinose (3.5%)	23	23.1	0.85	0.24	AA (< 0.5)	[50]
*B. succiniciproducens* JF4016	Batch	Pure xylose	–	7.67	4.6	0.60	0.80	FA (1.9), AA (2.6)	[51]
*A. succinogenes* 130Z	Batch	Corn stover (73.4%)	Glucose (10.1%), galactose (4.9%), arabinose (11.6%)	55.4	42.8	0.74	1.27	FA (-), AA (-)	[33]
*Y. lipolytica* PSA02004PP	*Batch*	*Pure xylose*	**–**	*60*	*11.2*	*0.19*	*0.13*	*AA (8.5)*	*This study*
*Y. lipolytica* PSA02004PP	*Batch*	*SCB (90.5%)*	Glucose (6.0%), arabinose (3.6%)	*37.8*	*5.6*	*0.13*	*0.09*	*AA (8.3)*	*This study*
*Y. lipolytica* PSA02004PP	*Fed-batch*	*Pure xylose*	**–**	*150*	*22.3*	*0.15*	*0.14*	*AA (25)*	*This study*

In all the studies mentioned above except current work, pH was controlled/maintained either by automatic addition of alkali agents or supplementing culture medium with pH regulators

Italic values represent the result obtained in this study

*AA* acetic acid, *FA* formic acid, *SSL*-spent sulphite liquor

^a^The yield is calculated on the basis of total sugars consumed

## Conclusions

The realization of biological SA production is highly dependent on utilization of low-cost renewable resources. The bio-based SA production from LCB can be a promising strategy as compared to the petrochemical route. The valorization of xylose is imperative for profitable and economical LCB-based SA production. *Y. lipolytica* can robustly metabolize a large variety of substrates including hydrophilic (glucose, glycerol, ethanol, acetate) as well as hydrophobic carbon sources (alkanes, fatty acids, oils), but it is unable to consume xylose, second major sugar in LCB. The current study made use of rational metabolic engineering strategy to develop a xylose-utilizing *Y. lipolytica* strain for manufacturing SA. The experimental results reported in this study demonstrate the promising potential of engineered strain to accumulate SA from pure as well as crude xylose. The accumulation of SA at low pH gives further advantage. In our knowledge, this is the first reported study utilizing metabolically engineered *Y. lipolytica* for SA production from xylose. The work serves as a proof of concept, and it creates room for further improvements for upcycling of agricultural residues into SA. In order to enhance the performance required for commercial production of SA, development of novel metabolic engineering strategies and process engineering work are required.

## Methods

### Materials used in this study

All chemicals used in this study were of analytical grade and purchased from Sigma Aldrich (USA) and Fisher scientific unless stated otherwise. All restriction enzymes, DNA ligase and Q5 Taq DNA polymerase used for the PCR and cloning were purchased from New England Biolabs (NEB) (USA). The xylose-rich lignocellulosic hydrolysate from SCB with following composition was obtained from Nova Pangea Technologies, UK. The composition of the hydrolysate was as follows (g/L): xylose, 42.8; glucose, 2.8; arabinose; acetic acid, 1.8 g/L; furfural < 1.0.

### Microorganism, culture maintenance and inoculum preparation

The current study made use of strain originated from adaptive evolution of engineered *Y.* *lipolytica* PSA02004 with deletion of *Ylsdh5* gene encoding a sub-unit of succinate dehydrogenase [18]. The recombinant *Y. lipolytica* strain was preserved in 20% glycerol (v/v) at − 80 °C and maintained on a petri dish containing YPD agar medium (1% yeast extract, 2% peptone, 2% dextrose and 2% agar) at pH 7.0 and 30 °C. The seed culture was grown in a 250-mL Erlenmeyer flask containing 50 mL minimal medium (see section Submerged cultivations in shake flask). The flasks for seed culture were inoculated by transferring a loopful of 48-h culture grown on a YPD plate. The final pH of the medium prior to sterilization was adjusted to 6.8. Cultivation was carried out for 24 h at 30 °C on a rotary shaker at an agitation speed of 250 rpm.

### Cloning and expression of heterologous xylose assimilation gene in *Y. lipolytica* strain

*Escherichia coli* (DH5α) strain was used for cloning and plasmid propagation. The strain was cultivated in the lysogeny broth (LB) liquid medium at 37 °C. The gene encoding xylose reductase (XR) (*YALI0D07634*), xylitol dehydrogenase (XDH) (*YALI0E12463*) and xylulokinase (XK) (*YALIF10923*) were extracted from the genome of Po1d using the appropriate primers (Additional file 1: Table S1) and the Golden Gateway (GG) assembly was constructed according to former studies [39, 40]. The GG was constructed with a scaffold of three genes comprising three transcription units and selection marker, flanked with integration targeting sequences, constructed on a destination vector backbone. Each gene was flanked with 396 nt of TEF promoter and 122 nt of Lip2 terminator sequences, both native to *Y.* *lipolytica*. *URA3* (1289 nt) gene was used as selection marker in this assembly. Random integrations in *Y. lipolytica* PSA02004 were driven through zeta sequences (305 nt and 395 nt for UP and DOWN, respectively). The expression vector was linearized using *Not*I enzyme and gel purified before transformation in *Y. lipolytica*. The overexpression cassette was transformed in the genome of *Y. lipolytica* using lithium acetate method described by Le Dall et al. [41]. The transformants were selected on YNBUra plates, the genomic DNA was isolated using the protocol developed by Lõoke et al. [42], and the positive transformants were identified with PCR. All the plasmids and strains used in this study are listed in Additional file 1: Table S2.

### Submerged cultivations in shake flask

The minimal medium used for fermentation had the following composition: xylose, 20 g/L; yeast nitrogen base (YNB), 1.7 g/L; NH_4_Cl, 1.5 g/L. The medium was prepared in 50 mM phosphate buffer. In case of co-fermentation with two carbon sources, each one was used at a level of 20 g/L. The initial pH was adjusted to 6.5–6.8 before inoculation by using 5 N NaOH. The submerged cultivations were carried out in 500-mL shake flasks containing 100 mL working volume. The flasks were inoculated with fresh inoculum at OD_600_ of 0.1 and kept at 30 °C under constant shaking at 250 rpm on a rotary shaker.

### Measurement of xylose reductase (XR) and xylitol dehydrogenase (XDH) activities

For measuring the enzymatic activities, cell-free extract was prepared. Initially, the cells were harvested by centrifugation at 8000*g* and 4 °C for 10 min. The cell pellet was then washed twice with 50 mM phosphate buffer (pH 7.2) and resuspended in the buffer. The cell disruption was performed in homogenizer by mixing the above-mentioned cells with 0.5 g (0.3 mm) glass beads and vortexed for 10 min. The homogenized mixture was centrifuged at 8,000*g* and 4 °C for 10 min, the supernatant was collected and used for quantifying enzyme activities. The protein concentration was determined by the Bradford method [43].

The activities of xylose reductase (XR) and xylitol dehydrogenase (XDH) were measured using a UV spectrophotometer (Jenway 6310, UK). The molar extinction coefficient of NADPH and NAD^+^ used for calculation is 6,220 m^−1^ cm^−1^. The XR activity was measured by the reduction of the coenzyme NADPH at 30 °C in a reaction medium consisting of 0.17 mM NADPH, 0.17 M xylose, 0.25 mg cell extract, and the final volume was made up to 0.5 mL using 0.1 M phosphate buffer. One unit of XR enzyme activity was defined as the amount of enzyme that catalyzed the oxidation of 1 μmol of NADPH per minute at 30 °C. The quantification of XDH activity was based on reduction of the coenzyme NAD^+^ at 30 °C. For XDH measurement, the reaction mixture consists of 1.5 mM NAD^+^, 0.15 M xylitol, 0.25 mg cell extract with a total volume of 0.5 mL made up by 0.1 M Tris buffer. One unit of XDH was defined as the amount of enzyme catalyzing the oxidation of 1 μmol of NAD^+^ per minute at 30 °C [44].

### Bioreactor studies

The batch experiments were performed in a 2.5-L bench-top bioreactor (Electrolab Bioreactors, UK) with 1.0-L working volume. The minimal medium with 60 g/L xylose was used for running bioreactor experiments. In case of lignocellulosic hydrolysate, the xylose concentration was 40 g/L. The temperature, agitation speed and aeration rate were controlled at 30 ℃, 600 rpm and 2.0 L/min, respectively. The starting pH was 6.8, and it remained uncontrolled during the fermentation. For fed-batch fermentations, the residual xylose concentration was maintained at or above 10 g/L with concentrated feed containing 500 g/L xylose and 5 g/L yeast extract.

### Analytical methods

The samples were withdrawn periodically and analyzed for OD_600_, pH, residual glucose, glycerol, xylose, xylitol, SA and AA. Cell growth was quantified by measuring the optical density at 600 nm wavelength in a 1-mm path-length cuvette using a double-beam spectrophotometer (Jenway 6310, UK). One unit of absorbance at 600 nm corresponded to a cell dry weight (CDW) of 0.21 g/L. The concentrations of glucose, glycerol, xylose, xylitol, SA and AA were measured by high-performance liquid chromatography (Agilent Technologies 1200 series, USA). The supernatants obtained by centrifugation of the culture samples at 10,000*g* for 10 min were filtered through a 0.22-µm PVDF membrane (Sartorius, Germany)) and eluted using Rezex ROA-Organic Acid H + (Phenomenex, USA) column at 60 °C attached with refractive index detector (RID) and diode array detector (DAD). The mobile phase and flow rate were 0.5 mM H_2_SO_4_ and 0.4 mL/min, respectively. All measurements were conducted in triplicates and the values were averaged. The standard deviation was no more than 10%.

## Supplementary information

**Additional file 1: Table S1.** Primers used in this study. **Table S2.** List of Plasmids and strain used in this study.

## Data Availability

All data generated or analyzed during this study are included in the manuscript.
