# Supplementary material for: Bioproduction of succinic acid from xylose by engineered Yarrowia lipolytica without pH control
Source: Biotechnol Biofuels. 2020 Jun 27;13:113. doi: 10.1186/s13068-020-01747-3 (PMC7321536; doi:10.1186/s13068-020-01747-3)
Supplement: Supplementary file 1 — Additional file 1: Table S1. Primers used in this study. Table S2. List of Plasmids and strain used in this study. [file 13068_2020_1747_MOESM1_ESM.docx]

**Table S1**: Primers used in this study

| Primer names | Sequence |
| --- | --- |
| XDH fw | ATGTCTTCTAACCCGTCATTTGTTCTTCGA |
| XDH rev | CTACTCCTCCTCGGGACCGTCAAT |
| XR fw | ATGTCCTTCAAGCTCGCCTCCG |
| XR rev | TTAGGCGAAAATGGGAAGGTTAGCGTA |

**Table S2**: List of Plasmids and strain used in this study

| **Plasmids or Strains** | **Short description** | **Reference or source** |
| --- | --- | --- |
| **Plasmids** |  |  |
| GGE0106 | Golden Gate three‐gene assembly, composed of *Y. lipolytica* xylulokinase (*XK*), xylitol dehydrogenase (*XDH*) and xylose reductase (*XR*) genes (ZUpNotI_*URA*_P1*Tef*_*XDH*_T1*Lip2*_P2T*ef*_*XR*_T2*Lip2*_P3*Tef*_*XK*_T3*Lip2*_ZDNotI). | [38] |
| **Strains** |  |  |
| *E. coli*  DH5α | Commercial transformation host for cloning | NEB |
| *Y. lipolytica* PSA02004 | Engineered strain of *Y. lipolytica* in which the *Ylsdh5* genes encoding succinate dehydrogenase, was deleted and subjected for adaptive evolution | [18] |
|  |  |  |
| *Y. lipolytica* PSA02004PP | *Y. lipolytica* PSA02004 integrated with the plasmid GGE0106 | In this study |
|  |  |  |
